# The Role of Biomarkers in the Diagnosis and Prognosis of Different Stages of Melanoma

**DOI:** 10.7759/cureus.38693

**Published:** 2023-05-08

**Authors:** Jane N Nwafor, Beatrice E Torere, Evelyn Agu, Lateef Kadiku, Tolulope Ogunyemi, Precious A Akinsanya, Omoniyi O Araromi, Darlington E Akahara, Okelue E Okobi

**Affiliations:** 1 Internal Medicine, The University of District of Columbia, Silverspring, USA; 2 Internal Medicine, North Mississippi Medical Center, Tupelo, USA; 3 Biology, University of Texas, Arlington, USA; 4 General Medicine, University of Lagos, Langhorne, USA; 5 Health Sciences, UnitedHealth Group, Minnetonka, USA; 6 Oncology, Holy Name Medical Center, Teaneck, USA; 7 Pathology and Laboratory Medicine, University of Ibadan, University College Hospital, Ibadan, NGA; 8 Medicine, Windsor University School of Medicine, Cayon, KNA; 9 Family Medicine, Medficient Health Systems, Laurel, USA; 10 Family Medicine, Lakeside Medical Center, Belle Glade, USA

**Keywords:** circulating tumor cells (ctcs), micrornas (mirnas), s100b, mia, biomarkers, melanoma

## Abstract

*Melanoma* is a skin cancer arising from melanocytes, the cells responsible for synthesizing melanin pigment, which gives the skin its color. Early diagnosis and treatment of melanoma increase survival rates. Clinical examination and biopsy are the primary tools used to diagnose melanoma. However, distinguishing between pre-malignant melanocytic lesions and early invasive melanoma histopathologically remains challenging. Therefore, additional modalities such as a detailed clinical history, imaging, genetic testing, and biomarkers have been applied to diagnose melanoma. This review discusses the current trends in biomarker advancements over the last 10 years to assist in the early detection and diagnosis of melanoma. Biomarkers such as melanoma-associated antigens (MAAs), S100B, microRNAs (miRNAs), and circulating tumor cells (CTCs) have the potential to aid in the detection, diagnosis, and prognosis of melanoma. However, the application of biomarkers in the diagnosis of melanoma is still evolving.

## Introduction and background

Melanoma is an aggressive skin cancer that develops from melanocytes, the cells that produce pigment in the skin. It remains the deadliest form of skin cancer [1-3]. However, melanoma is potentially curable with early diagnosis and treatment [1-4]. In the US, it is the 5th most common cancer and the deadliest form of skin cancer, accounting for about 80% of deaths related to skin cancer. Its incidence has continued to rise since the 1970s, with over 1 million people living with melanoma. It also accounts for 1.7% of global cancers, with increasing incidence in developed countries [1-6]. Although the 5-year relative survival rate has increased to 93.7%, survival for advanced-stage disease remains relatively low (29.8%) [1-4]. Several factors have contributed to this increase in overall survival, including advances in diagnostic approaches and targeted and immune therapies. Common risk factors include fair-skinned populations and regions of lower latitude [1-3]. It is also more common in older individuals, with an average age of diagnosis of 65, and in men [1-3].

Diagnosing melanoma can be challenging because it can present in various ways, such as a changing or new mole, a spot or bump that looks different from other spots on the skin, or a sore that does not heal. Also, the various cytomorphologic presentations of melanoma pose an immuno-histologic challenge [4-8]. This is because its immunohisto-markers may resemble those of other tumors such as germ cell tumors, neuroendocrine, and other carcinomas. Clinical examination and biopsy are the main tools used to diagnose melanoma [1]. However, even with a biopsy, distinguishing between a benign mole and a melanoma can be challenging for clinicians [2-8]. Therefore, additional imaging and genetic testing have been necessary for a more objective diagnosis [2-6,8,9-15].

While clinical examination and biopsy are the gold standards for melanoma diagnosis, the challenges in distinguishing between a benign mole and a melanoma highlight the need for additional tests to aid in diagnosis [1-4,7-12]. Early detection and prevention through sun protection are critical in reducing the morbidity and mortality associated with melanoma. Therefore, identifying biomarkers associated with the disease has therapeutic and prognostic implications, particularly in advanced-stage melanoma, where early diagnosis and treatment can lead to high survival rates [1-3, 16-31]. In this article, we will discuss current methods of melanoma diagnosis, including clinical markers, imaging, and histopathological examination. We will also explore the potential roles some biomarkers play in contributing to melanoma diagnosis and prognosis. This paper, therefore, aims to provide an overview of the current approaches to melanoma diagnosis using some biomarkers.

Methodology

**Figure 1:**
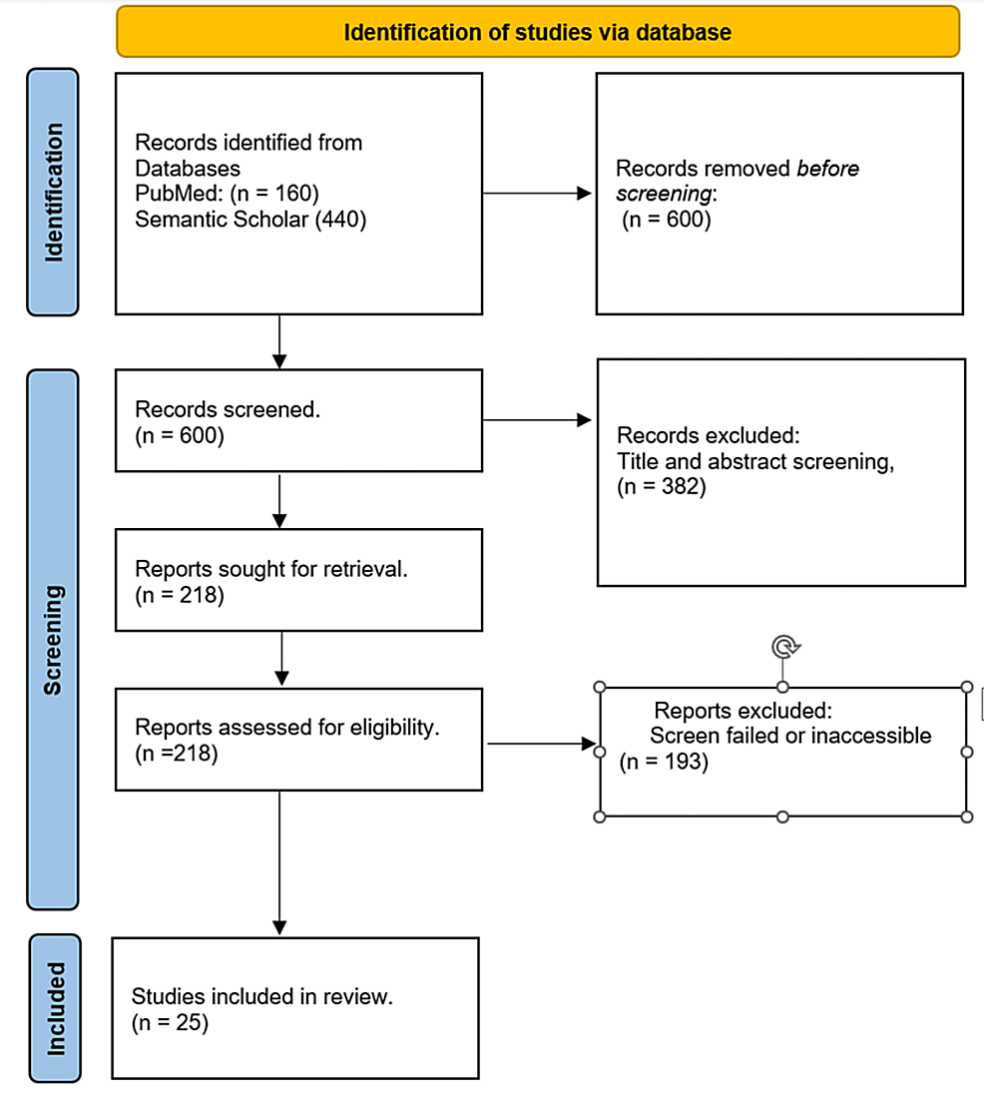
Prisma flow diagram of inclusion and exclusion. Ref no-  [5-39]

To review the current place for biomarkers in melanoma diagnosis over the last 10 years, articles related to melanoma and biomarkers were searched for in PubMed. The mesh terms "melanoma," "biomarkers," "diagnosis," and "prognosis" were deployed using the Boolean operator "and/or". The search filter was set to capture articles published in the last 10 years (2013-2023). Then abstracts and free full texts were selected. Articles published on clinical trials, meta-analyses, and randomized controlled trials were selected. In addition, the selected search criteria were set to generate papers done by humans and written in English. The generated abstracts were screened further to select papers that captured the objective and focus of this manuscript. Then the article content was reviewed for appropriateness with the objective of the paper. The inclusion and exclusion criteria are listed in Table 1 below. The Prisma flow for the selected studies is seen in Figure 1 below. 

**Table 1:** Inclusion and exclusion criteria.

Inclusion criteria	Exclusion criteria
1) Literature relevant to the role of biomarkers in the diagnosis or prognosis of melanoma	1) The studies that did not discuss biomarkers and melanoma were excluded because the study's objective was focused on the role of biomarkers in melanoma
2) The studies must be original case-control studies, cohort studies, or randomized clinical controlled trials.	2) Sccoping reviews or other research types that weren't data-driven were excluded.
3) The selected studies may be heterogeneous but must have measurable endpoints.	3) Qualitative studies were excluded
4) Human studies.	4) Human studies.
5) The studies must be published in a peer-reviewed journal to maintain validity and reliability.	5) The studies published in non-peer-reviewed journals and dissertations were excluded.
6) The studies must be originally published in English for readability by the reviewers.	6) The studies originally published in a language other than English were discarded.
7) Works of literature published between 2013 and 2023	

## Review

Current methods of melanoma diagnosis

Clinical Markers

The first step in melanoma diagnosis is the recognition of atypical lesions [1-3,6-12-15]. The ABCDE method is a straightforward acronym designed to assist the general public and medical professionals identify potential melanomas based on their characteristics. The letters represent five important characteristics of an aberrant skin lesion: asymmetry, irregular border, color variability/change, diameter, and progression. These characteristics are frequently associated with melanomas that are early or in situ. Asymmetry refers to the irregularity in the lesion's shape, in which one half differs from the other. Border irregularity refers to the blurring, notching, or unevenness of the lesion's border. Color variability/change refers to the lesion having a range of colors, such as various hues of brown or black, or gradually becoming darker or paler. Diameter refers to the lesion's extent, typically greater than 6 millimeters. Changes in the lesion's size, shape, color, or texture over time are referred to as evolving/evolution [1,6-8,14,15]. Once a lesion is identified as potentially malignant, a biopsy is performed, and the tissue is analyzed under a microscope to confirm the diagnosis [1-8]. Dermoscopy can be used to improve the accuracy of tissue sampling [1-3,6,8,12-16]

Imaging

In addition to clinical markers, imaging techniques such as ultrasonography, magnetic resonance imaging (MRI), and computed tomography (CT) scans can aid in diagnosing melanoma. Ultrasonography is useful for assessing the thickness of melanomas, while MRI and CT scans can provide detailed images of the internal structures of the skin and surrounding tissue, as well as rule out metastasis to other organs [1,3-8, 16-23]. No baseline investigations are recommended for patients with stage 0-II cutaneous melanoma as per AAD and stage 0 to IIIB as per NCCN. ESMO and NCCN recommend whole-body positron emission tomography (PET) and brain magnetic resonance imaging (MRI) for patients with stage III and higher disease. ESMO recommends PET and MRI of the brain for patients with tumor pT3b and higher. Additionally, as per NCCN, PET and MRI brain should be considered for patients with early-stage disease and signs and symptoms of metastatic disease and patients with the high-risk disease for patients such as those who present with positive sentinel lymph node or microscopic satellite or in-transit metastatic lesions on pathology or with clinically palpable lymph nodes [24]. However, CCA does not recommend imaging for patients with positive sentinel lymph nodes (Grade B) while recommending PET and MRI brain imaging for palpable lymph nodes (Grade B) [24].

Histopathological Examination

The gold standard for melanoma diagnosis is histopathological examination. A pathologist examines the biopsy specimen under a microscope to determine if the lesion is malignant [1-8]. A typical melanoma under a tissue microscope can be described using several descriptions. When a pathologist examines a melanoma under a microscope, they typically look for several defining characteristics of the tumor. For example, melanocytic cells disposed of in sheets and nests and the presence or absence of perineural invasions. Other e characteristics include the number of lymphocytes, or TILs (tumor-infiltrating lymphocytes), present within the lesion. The presence of TILs can indicate that the immune system has recognized the melanoma cells as abnormal and is attacking them [25-32]. The pathologist may describe the TILs as "brisk," "non-brisk," or "absent," and they may also use the terms "mild" or "moderate" [1,3,5-6,8]. Other defining characteristics of melanoma under a tissue microscope include the type of melanoma, the depth of invasion, the presence or absence of ulceration, the mitotic count, the presence or absence of regression, and the presence or absence of satellite lesions. The pathologist may also look at the specimen type, the procedure used to remove the lesion, the site and side of the body where the lesion was located, melanoma subtype, excision margin, size of the tumor, and whether the tumor is in situ or invasive [4-6].

Biomarkers for Melanoma Diagnosis

**Figure 2:**
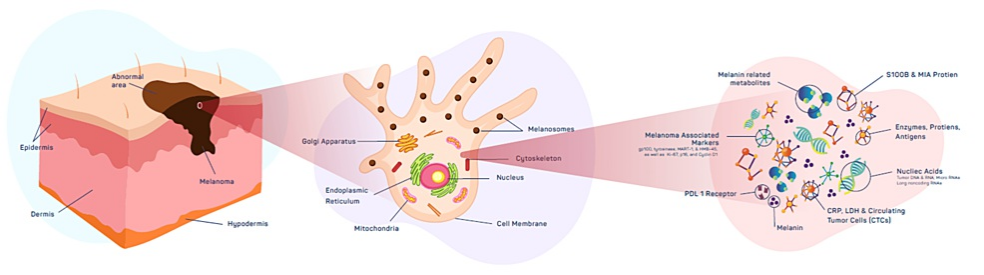
The above is an illustration of melanoma with some of its associated markers and products of tumorigenesis. The illustration above is an original conception and creation of the authors.

Early melanoma molecular characteristics include genetic and cellular structural modifications, such as abnormalities in collagen-like sequences or structural proteins, oncogenic BRAFV600E mutations, UV-induced DNA mutations, molecular signaling pathways, or UV-induced DNA mutations. The expression of chemokines, cytokines, endopeptidases, phaeomelanin precursors, melanin-associated antigens, dimeric proteins like S100-B, RNA/DNA microarrays products, and other tumorigenic byproducts may occur as a result of these complex early or late molecular changes and processes in a cell. Overall patient survivorship and prognosis continue to be influenced by early melanoma identification and therapy [1-3,6-9,15]. Biomarkers are molecules that may be detected in blood, tissue, or other biological samples and can provide diagnostic or prognostic information about the presence or development of disease [6-12]. Melanoma-associated antigens (MAAs), microRNAs (miRNAs), S100B, CRP, LDH, and circulating tumor cells (CTCs) are possible biomarkers for the diagnosis of melanoma [4,6-12]. Other hundreds of additional potential melanoma biomarkers have also been discovered and extensively researched in published literature. These include Melan-A, circulating tumor DNA, cell-free DNA, and melanoma-inhibitory antigens (MIAs) [5-14,18-25]. With no single biomarker currently matching the requirements for a minimum useful test (ctDNA), the present state of biomarkers for melanoma detection is still in its infancy [6-14,18-25]. These distinctive markers will be discussed (see conceptual illustration in Figure 2 below).

Framework for determining minimally useful biomarker test characteristics

The framework for determining the minimally useful biomarker test characteristics involves the assessment of the sensitivity, specificity, positive predictive value (PPV), negative predictive value (NPV), and area under the receiver operating characteristic curve (AUC-ROC) [17,18].

MAAs (Pmel-17/gp100 and MART‐1/Melan-A) are proteins specifically expressed by melanoma cells, not normal cells. They have been extensively studied as potential biomarkers for melanoma due to their ability to elicit an immune response and their specificity to melanoma cells. The expression of MAAs has been shown to correlate with disease progression and prognosis in melanoma patients [5-8]. Some other studies [9] investigated the expression of several MAAs in melanoma patients and found that the expression of one MAA, MAGE-A3, was significantly associated with poor survival outcomes [4-11-14]. Prognostically, patients with high levels of MAA (CYT-MAA) had an 81% risk of recurrence compared with patients with undetectable levels. In a study to evaluate the prognostic significance of MAA in 117 patients, Irene et al. concluded that MAA is a significant prognostic biomarker, especially in resected patients [31]. The role of Melan-A has also been studied over the last 10 years and reported conflicting outcomes. Some studies have reported that specific T-cell-recognized membrane proteins (Melan-A) have also demonstrated high sensitivity (about 93%) and specificity (about 99%) in differentiating non-melanocytic cells from melanoma, especially for primary tumors. Other authors, on the contrary, reported lower sensitivity (about 86%) and expressed concerns about its specificity because of its ability to stain other pigmented epithelium like the retina and non-pigmented epithelium or their derivatives like Leydig, adrenocortical, ovarian, and theca cells [26]. It has also been demonstrated that in some situations, the absence of MAA or HLA expression in a subset of lesions correlates with rapid regression. Although the precise mechanism of the relationship is not yet completely understood [32-34].

miRNAs are small non-coding RNA molecules that regulate gene expression by targeting mRNA molecules. They have been identified as potential biomarkers for various cancers, including melanoma. Several studies have investigated the expression of miRNAs in melanoma and their potential use as diagnostic and prognostic biomarkers. For example, some authors have identified several miRNAs that were differentially expressed in melanoma patients compared to healthy controls, suggesting their potential use as diagnostic biomarkers [6-15,33]. Some review articles published have suggested the potential use of other biomarkers, including microRNAs and exosomes, for diagnosing melanoma [26-33]. The authors suggested that these biomarkers may potentially improve the accuracy of melanoma diagnosis, as well as predict disease progression and response to therapy [4-15]. In a study analyzing 126 samples from people with melanoma, miRNA levels significantly changed at more advanced stages of cancer [34]. Other studies have also shown some hope in the role of miRNA as a prognosticating molecule for progression. For example, although their result was inconclusive, in a pooled meta-analysis of 2669 patients, Shanthi et al. made similar observations with an overall effect size of 1.043 (95% CI 0.921-1.181; p = 0.506) and the likelihood of death of patients with this marker at 4.3% [35]. 

CTCs are cells shed from a primary tumor and enter the bloodstream. They have been studied as potential biomarkers for various cancers, including melanoma, due to their ability to provide information about tumor metastasis and treatment response. Several other studies have investigated the presence of CTCs in melanoma patients and found that the presence of CTCs was associated with poor prognosis and disease progression [4-15]. In addition, several other studies have associated CTC with poor prognosis. For example, in a meta-analysis of 5433 patients with melanoma from 53 studies to determine the prognostic value of CTC, Morcelin et al. [36] determined that positivity rates in early-stage melanoma were better than late-stage, with 2.45 and 2.42 for progression-free survival and overall survival, respectively.

Biomarkers, such as circulating tumor DNA (ctDNA) and cell-free DNA (cfDNA), have emerged as potential diagnostic tools for melanoma. Some studies [6-10] demonstrated the potential of ctDNA as a biomarker for detecting melanoma. A study enrolled 135 patients with advanced melanoma, and ctDNA was detected in 57% of the patients [13]. The researchers found that ctDNA was associated with tumor burden, stage of disease, and overall survival. Furthermore, ctDNA was detected in patients with smaller tumors and earlier stages of the disease, suggesting that ctDNA may be useful for detecting melanoma at an earlier stage [13-15]. The researchers concluded that ctDNA may be a useful tool for diagnosing melanoma, especially in cases where traditional diagnostic methods, such as biopsy, are impossible. 

MIA is a secreted protein overexpressed in melanoma cells and has been shown to have diagnostic and prognostic value [4-15,37-39]. However, its sensitivity and specificity are suboptimal, and it has limited utility as a standalone biomarker. According to a study, the diagnostic value of the Melanoma Inhibitory Activity (MIA) serum marker was investigated in the follow-up of patients with stage I or II cutaneous melanoma. The study included 5,334 MIA serum values from 1,079 consecutive melanoma patients in stages I and II obtained during routine follow-ups at scheduled intervals. The sensitivity and specificity of MIA were calculated, and the area under the receiver-operating characteristics curve and Somers' Dxy rank correlation were assessed. The study found that metastasis occurred in 137 patients with a sensitivity of MIA testing of 67.6% in stage I and 65.6% in stage II patients. The specificity was 76.9% for stage I and 66.7% for stage II patients. In that study, the most reliable normal upper limit for MIA was redefined at 12.0 ng/ml when compared with 8.8 and 15.0 ng/ml.

Furthermore, multivariate analysis revealed significantly more frequent false-positive values in elderly women and men with an increased Breslow thickness. The study further reported that MIA levels increased in 5.6% of individuals with early-stage melanoma and up to 89.5% of people with late-stage melanoma [25]. ctDNA is a promising biomarker that can be detected in the blood of melanoma patients [4-6,8-15,20-25]. However, its diagnostic performance is still uncertain and requires further validation in clinical trials.

S100B protein is a calcium-binding protein expressed in melanoma cells and proposed as a diagnostic biomarker [5-8,11-15,39]. However, its diagnostic accuracy is affected by several factors, such as age, sex, and tumor thickness. According to a quantitative analysis of data to compare LDH and S100 B's prognostic and discriminative ability for melanoma, six eligible studies, which included 1,033 patients with cutaneous melanoma, serum S100B showed significantly greater discriminative ability in identifying disease relapse [pooled area Under the ROC (AUROC) 78.64 (95% CI 70.28; 87.01)] compared to serum LDH [AUROC 64.41 (95% CI 56.05; 72.78)] (p=0.013). In the risk of death analysis, ten eligible studies with 1,987 patients were included. The prognostic performance of serum S100B [pooled estimate of adjusted hazard ratio (HR) 1.78 (95% CI 1.38; 2.29)] was independent but not superior to that of serum LDH [HR 1.60 (95% CI 1.36; 2.29)] [30].

HMb-45 has also been explored over the last century as a potential immunohistochemical marker. This monoclonal antibody that stains glycoproteins (gp100, Pmel17) between the junctional nevus cells and melanoma has a sensitivity of between 66 and 97%, especially in a primary lesion, with a lesser sensitivity for its metastatic variant. Its specificity has also been reported in various published works to range between 91-100% in diagnosing melanoma, but unfortunately, showing a poor ability to detect its desmoplastic variant [26].

Elevated levels of lactate dehydrogenase (LDH) have been studied over the years to correlate with predictors of overall survival in melanoma. Although it has yet to be quantitively defined, the utility of the level of LDH change from baseline in predicting overall survival (OS) has been studied over the years. Several studies and guidelines have corroborated the significance of this marker. In a 10-year retrospective study to analyze the predictive value of circulating serum biomarkers in 48 patients, Arana et al. correlated the change from baseline as a predictor of OS [38]. In another study to compare the diagnostic and predictive value of a combination of comparative serum biomarkers (S100B, LDH, MIA, and proteasome) in 121 patients, Henry et al. established positive correlative values between these markers and OS [39]. The 7th AJCC recommended the use of elevated LDH to categorize metastatic lesions. However, this recommendation was modified in the 8th edition to include anatomic sites for M1C metastatic category [37]

Recently, utilizing non-invasive methods to analyze melanotic gene expression from skin lesions has shown remarkable success. In several studies evaluating the presence of gene expression, the non-invasive biopatch collection method has demonstrated valuable success. For example, in their study, Gerami and his research group demonstrated that such a non-invasive method improved biopsy sensitivity from 95.0% to 98.6% and specificity from 32.1% to 56.9%. Some other studies have also reported similar improvements in sensitivity and specificity of using these non-invasive methods of collecting and analyzing melanotic gene expression; 91% and 69% sensitivity and specificity, respectively [27-29]. The seemingly non-invasive technology has certain limitations, however, including the inability to detect melanotic lesions that are present on the mucosa, nails, soles of the feet, or palms, as well as the fact that its predictive value is not yet properly understood [29].

Future directions in melanoma diagnosis

Currently, skin cancers, including melanoma, are primarily diagnosed visually, which lends itself to multiple imaging modalities such as naked-eye clinical images, dermoscopy, reflectance confocal microscopy (RCM), and optical coherence tomography (OCT) [1-3]. As for future directions in melanoma diagnosis research, there is a need to develop more accurate and non-invasive diagnostic techniques. One area of research is using artificial intelligence (AI) to analyze clinical and histopathological images to aid in diagnosis and prognosis [1-3,28-34]. Additionally, there is a growing interest in developing liquid biopsies to detect circulating tumor cells and cell-free DNA, which may help with early detection and monitoring of treatment response [1-3,17-26]. Most of these available molecules had higher serum levels in more advanced melanoma stages, and hence, these indicators play a minimal role in early diagnosis [33]. Although a few other markers have shown promising findings as potential prognostic indicators for the early diagnosis of disease development or the prognosis of therapeutic outcomes, this population of patients at risk for melanoma may thus benefit from study designs focused on choosing more sensitive and specific markers for early diagnosis. These advancements in research may aid in the early detection and monitoring of treatment response, ultimately improving patient outcomes.

Study limitation

Selection bias may have played a role in this study due to the arbitrary selection of a few significant biomarkers and the absence of all available markers.

## Conclusions

In conclusion, melanoma is a type of skin cancer that develops when skin cells called melanocytes grow out of control. Early melanoma detection and diagnosis are essential for successful treatment and improved patient outcomes. Several biomarkers have been identified as potential diagnostic tools for melanoma, and various methods and technologies for detecting these biomarkers exist. The use of biomarkers for melanoma diagnosis is an active research area, with several promising biomarkers under study. Overall, there is ongoing research into potential biomarkers for melanoma diagnosis, but more work is needed to validate these biomarkers and determine their clinical relevance. Their diagnostic performance must be validated in large-scale clinical trials before being used in clinical practice. The framework for determining the minimally useful biomarker test characteristics provides a standardized approach for evaluating the diagnostic accuracy of biomarkers, ensuring that only biomarkers with high diagnostic accuracy are used in clinical practice.

## Appendices

All authors played contributory roles that overlapped, such as: conceptualization, design, cross-referencing, and fact-checking, formal analysis and interpretation of data, project administration, curation, visualization, writing - original draft, writing - review & editing, Supervision, oversight, and leadership, correspondence, data curation, consenting, quality control, internal review, communications, data collection and archiving, software, literature search, validation, and approval.
